# Functional Expression of All Human Sulfotransferases in Fission Yeast, Assay Development, and Structural Models for Isoforms SULT4A1 and SULT6B1

**DOI:** 10.3390/biom10111517

**Published:** 2020-11-06

**Authors:** Yanan Sun, David Machalz, Gerhard Wolber, Maria Kristina Parr, Matthias Bureik

**Affiliations:** 1 School of Pharmaceutical Science and Technology, Health Sciences Platform, Tianjin University, Tianjin 300072, China; suny72@zedat.fu-berlin.de; 2 Pharmaceutical and Medicinal Chemistry (Pharmaceutical Analyses), Institute of Pharmacy, Freie Universitaet Berlin, 14195 Berlin, Germany; 3 Pharmaceutical and Medicinal Chemistry (Computer-Aided Drug Design), Institute of Pharmacy, Freie Universitaet Berlin, 14195 Berlin, Germany; david.machalz@fu-berlin.de (D.M.); gerhard.wolber@fu-berlin.de (G.W.)

**Keywords:** drug metabolism, phase II, proluciferin, sulfation, sulfotransferase

## Abstract

Cytosolic sulfotransferases (SULTs) catalyze phase II (conjugation) reactions of drugs and endogenous compounds. A complete set of recombinant fission yeast strains each expressing one of the 14 human SULTs was generated, including SULT4A1 and SULT6B1. Sulfation of test substrates by whole-cell biotransformation was successfully demonstrated for all enzymes for which substrates were previously known. The results proved that the intracellular production of the cofactor 3′-phosphoadenosine 5′-phosphosulfate (PAPS) necessary for SULT activity in fission yeast is sufficiently high to support metabolite production. A modified variant of sulfotransferase assay was also developed that employs permeabilized fission yeast cells (enzyme bags). Using this approach, SULT4A1-dependent sulfation of 1-naphthol was observed. Additionally, a new and convenient SULT activity assay is presented. It is based on the sulfation of a proluciferin compound, which was catalyzed by SULT1E1, SULT2A1, SULT4A1, and SULT6B1. For the latter two enzymes this study represents the first demonstration of their enzymatic functionality. Furthermore, the first catalytically competent homology models for SULT4A1 and SULT6B1 in complex with PAPS are reported. Through mechanistic molecular modeling driven by substrate docking, we pinned down the increased activity levels of these two isoforms to optimized substrate binding.

## 1. Introduction

The plethora of biotransformations that together constitute human drug metabolism is subdivided into phase I (functionalization) and phase II (conjugation) reactions. The latter are carried out by Uridine 5′-diphospho-glucuronosyltransferases (UGTs), sulfotransferases, glutathione transferases, and other enzymes [1]. Cytosolic sulfotransferases (SULTs) catalyze the transfer of a sulfate group from the universal sulfate donor 3′-phosphoadenosine 5′-phosphosulfate (PAPS) to both endogenous and xenobiotic compounds [2]. In these reactions, the SO_3_^−^ moiety is transferred to hydroxy or amino functions of small molecule substrates. While the majority of known sulfations lead to metabolites with reduced biological activity compared to their parental compounds, there are also some examples of metabolic activation by this process [3]. There are 14 human SULTs that belong to the families SULT1, SULT2, SULT4, and SULT6, respectively, whereas SULT3 and SULT5 family members are not present in humans [4]. Most of the human SULTs are reported to be functionally expressed in *Escherichia coli* [5] or in *Saccharomyces cerevisiae* [6]. For two of these enzymes, namely, SULT4A1 and SULT6B1, no endogenous substrate nor activity data were ever reported before this study. Human SULT4A1 was originally identified in brain tissue [7] and displays a very high level of evolutionary conservation across species. More than a decade ago it was suggested that this enzyme might not be functional because it lacks part of one of the PAPS binding regions [8]. However, a severe phenotype and early postnatal death in SULT4A1 knock-out mice very recently revealed that SULT4A1 is an essential neuronal protein at least in this species [9]. Human SULT6B1 was first identified in 2004 [10] and belongs to a family that is also well conserved from mammals to fish, birds, and amphibians. In SULT6 enzymes, the sulfotransferase dimerization motif KXXXTVXXXE [11] is not retained, which suggests that they exist as monomers [2]. Human SULT6B1 is mainly expressed in the testes [10], whereas its mouse homologue is expressed in a variety of tissues [12]. The latter was reported to metabolize thyroxine and bithionol [12]. Previously, we successfully used fission yeast *Schizosaccharomyces pombe* for the functional expression of orphan cytochrome P450 (CYP) enzymes, such as CYP2A7, CYP4Z1, CYP4A22, and CYP20A1 [13,14,15,16], and UGTs such as UGT1A5 [17]. Homology models of these understudied enzymes guided by substrate activity data helped to increase knowledge on their functionality. There are two endogenous CYPs but no UGT or SULT homologues in fission yeast [18]. The aims of the present study were to evaluate fission yeast as a host for the recombinant expression of human SULTs and to assess its suitability for the functional production of SULT4A1 and SULT6B1. Furthermore, we aimed to rationalize our experimental results by modeling SULT–substrate complexes using homology modeling and substrate docking experiments.

## 2. Materials and Methods

### 2.1. Chemicals and Reagents

Na_2_HPO_4_, NH_4_Cl, glucose, KH_2_PO_4_, NH_4_I, and potassium hydrogen phthalate were from Chemart Chemical (Tianjin, China). MgCl_2_ 6H_2_O, CaCl_2_ 2H_2_O, KCl, Na_2_SO_4_, nicotinic acid, inositol, sodium pantothenate, biotin, MnSO_4_, ZnSO_4_ 7H_2_O, FeCl_3_ 6H_2_O, KI, CuSO_4_ 5H_2_O, H_3_BO_3_, MoO_4_ 2H_2_O, citric acid, agar, and thiamine were from Kermel Chemical (Tianjin, China). Triton-X100 was from Leagene (Beijing, China). NH_4_HCO_3_ was from Jiangtian Chemical (Tianjin, China). Tris-HCl was from AKZ-Biotech (Tianjin, China). E.Z.N.A. Plasmid Mini Kit and E.Z.N.A. Cycle Pure Kit were from Omega Bio-tek (Norcross, GA, USA); *E. coli* cells were from General Biosystems (Anhui, China); 4-nitrophenol, 1-naphthol, 7-hydroxycoumarin, and dehydroepiandrosterone (DHEA) were from Accela ChemBio Co., Ltd. (Shanghai, China); UGT-Glo substrates A (GSA, 6-hydroxy-4-methylbenzo[*d*]thiazole-2-carbonitrile) and B (GSB, 6-((3-aminobenzyl)amino)benzo[*d*]thiazole-2-carbonitrile) were from Promega (Madison, WS, USA); S9 fractions of human liver cells were from Sekisui XenoTech (Kansas City, KS, USA). All other chemicals and reagents used were of the highest grade available.

### 2.2. Fission Yeast Media and General Techniques 

General DNA manipulation methods were performed using standard techniques [19] and the preparation of media and basic manipulation methods of *S. pombe* were carried out as described [20]. Briefly, strains were generally cultivated at 30 °C in Edinburgh minimal medium (EMM) with supplements of 0.1 g/L final concentration as required. EMM was prepared with NH_4_Cl (93.5 mM), glucose (2% *w*/*v*), Na_2_HPO_4_ (15.5 mM), potassium hydrogen phthalate (14.7 mM), and a given amount of a salt, vitamin, and mineral solution. Liquid cultures were kept shaking at 150 rpm. Thiamine was used at a concentration of 5 μM throughout.

### 2.3. Expression Plasmid Construction 

Synthetic cDNAs encoding for each of the 14 human SULTs were synthesized by General Biosystem (Anhui, China) and cloned into both the integrative vector pCAD1 [21] and the replicating vector pREP1 [22] for expression in fission yeast. Both expression vectors contain the thiamine-repressible nmt1 promotor which allows for strong expression in *S. pombe* [23]. The correctness of all expression constructs was confirmed by automated sequencing.

### 2.4. Fission Yeast Strain Construction 

Transformation of fission yeast was done using the lithium acetate method [24]. Briefly, strain NCYC2036 (genotype *h^-^ ura4-D18*) [25] was transformed with pCAD1-SULT expression constructs to yield a set of new strains which contained the SULT genes integrated into the *leu1* locus on chromosome II of fission yeast under control of the nmt1 promotor [23]. Correct chromosomal integration of the pCAD1 constructs into the *leu1* locus was confirmed by replica plating colonies on EMM lacking leucine. These 14 new strains were in turn transformed with the corresponding pREP1-SULT plasmids.

### 2.5. Whole-Cell Biotransformation in Shaking Flasks

Whole-cell biotransformation was essentially done as described previously [6]. Briefly, wet fission yeast cells were suspended at a concentration of 25% (*w*/*v*) in 100 mM potassium phosphate (KPi) buffer (pH 7.4) containing 1% (*w*/*v*) ammonium sulfate, and 8% (*w*/*v*) glucose. Substrate stock solution (100 mM in DMSO) was added to the cell suspension to a final concentration of 1 mM. Biotransformation was performed at 30 °C with shaking at 150 rpm for the times indicated. Afterwards, three volumes of acetonitrile were added to the reaction solution. After centrifugation at 10,000× *g* for 10 min, the supernatant was evaporated and the remaining pellet resolved in 50 μL of the solvent for the HPLC analysis.

### 2.6. Biotransformation with Enzyme Bags

This was essentially done as described in [17] with slight modifications. Briefly, fission yeast strains were grown in 10 mL liquid culture of EMM with supplements as needed at 30 °C and 230 rpm for 24 h. For each assay, 5 × 10^7^ cells were transferred to 1.5 mL Eppendorf tubes, pelleted, and incubated in 1 mL of 0.3% Triton-X100 in Tris-KCl buffer (200 mM KCl, 100 mM Tris-Cl pH 7.8) at room temperature for 60 minutes at 150 rpm to allow permeabilization. Cells were then washed thrice with 1 mL of NH_4_HCO_3_ buffer (50 mM, pH 7.8) and directly used for SULT-dependent reactions. Enzyme bags were resuspended in 200 µL of NH_4_HCO_3_ buffer (50 mM, pH 7.8) containing 100 µM PAPS and substrate as indicated. For luminescence assays, enzyme bags were resuspended in 30 µL assay buffer (containing 8 µL 5 * UGT-Glo buffer, 100 µM PAPS, and either 10 µM UGT-Glo substrate A (GSA) or 50 µM UGT-Glo substrate B (GSB) as indicated). Biotransformations were done for 3 h at 37 °C in a shaking incubator (1000 rpm). Afterwards, the reaction mixtures were transferred to 1.5 mL Eppendorf tubes and centrifuged at 16,000× g for 1 min. The supernatants were then either analyzed by LC–MS or transferred to white 96-well microtiter plates for luminescence measurements. 

### 2.7. HPLC–UV and LC–MS Analysis

Analytic instruments were composed of a micrOTOF focus mass spectrometer (BrukerDaltonics, Bremen, Germany) hyphenated by electrospray ionization (ESI) to a 1290 infinity II liquid chromatography system (Agilent, Santa Clara, CA, USA), equipped with a LiChrospher^®^100 reversed phase C18 column (5 µm, 4.0 × 125 mm, Merck KGaA, Darmstadt, Germany) or a Kromasil 100-5-C18 column (for analysis of 4-nitrophenol biotransformations, 5 µm, 4.6 × 250 mm, Akzo Noble, Arlöv, Sweden). Mobile phase flow rate was 0.5 mL/min. MS parameters were set using negative ion mode with spectra acquired over a mass range of *m*/*z* 50–1000; capillary voltage, +3500 V; drying gas temperature, 180 °C; dry gas flow, 6 L/min; nebulizing gas pressure, 1.2 bar. The accurate mass data of molecular ions was calculated using Bruker Compass Data Analysis 4.1 (BrukerDaltonics, Bremen, Germany). 

For the analysis of the biotransformations of 7-hydroxycoumarin and 4-methyl-7-hydroxycoumarin, the column compartment was maintained at 25 °C. Mobile phase A was water and mobile phase B was methanol. The following gradient mode was used: 0–2 min 10% B, 2–8 min 10–60% B, 8–10 min 60–10% B, 10–12 min 10% B. Detection was performed by UV at a wavelength of 320 nm.

For the analysis of 1-naphthol biotransformations the column was maintained at 25 °C. The mobile phase A was water with 0.2% acetic acid and mobile phase B was methanol with 0.2% acetic acid. The following gradient mode was used: 0–15 min 15–90% B, 15–18 min 90% B. Detection was performed by UV at a wavelength of 280 nm.

For the analysis of 4-nitrophenol biotransformations the column was maintained at 25 °C. The separation was performed with an isocratic mixture of water with 0.2% acetic acid and methanol (1:1, *v*/*v*) for 13 min. Detection was performed by UV at a wavelength of 280 nm.

For the analysis of DHEA biotransformations, the column was maintained at 35 °C. The mobile phase A was water with 0.1% formic acid and mobile phase B was 90% methanol and 10% water with 0.1% formic acid. The following gradient mode was used: 0–5 min 50% B, 5–10 min 50–100% B, 10–15% min 100% B. 

For the analysis of GSA biotransformations a Q Exactive^TM^ HF Combined Quadrupole Orbitrap Mass Spectrometer (Thermo Fisher, Waltham, MA, USA) and a Kromasil 100-5-C18 column (5 µm, 4.6 × 250 mm) were used. The column was maintained at 25 °C, and the flow rate was 0.5 mL/min. The mobile phase A was water and mobile phase B was methanol. The following gradient mode was used: 10–32 min 10–95% B, 32–35 min 95% B, 35–36 min 95–10% B, 36–40 min 10% B. 

### 2.8. Bioluminescence Detection

Supernatants from the SULT-dependent biotransformations were transferred to white microtiter plates and an equal amount of reconstituted luciferin detection reagent was added to each well. Plates were then incubated at room temperature for 20 min and luminescence was recorded on a Magellan infinite 200Pro microplate reader (Tecan; Männedorf, Switzerland). In all cases reaction parameters (reaction times and enzyme concentrations) were within the linear range. All measurements were done at least three times in triplicate.

### 2.9. Statistical Analysis

All data are presented as mean ± SD. Statistical significance was determined using a two-tailed *t*-test. Differences were considered significant if *p* < 0.05. Statistical analysis was done using GraphPad Prism 5.01 (GraphPad Software, Inc., La Jolla, CA, USA).

### 2.10. Homology Modeling for SULT4A1 and SULT6B1

No experimentally solved 3D structure of the catalytically competent complex with cofactor PAPS exists for either SULT4A1 or SULT6B1. Hence, structural homology modeling was conducted on the I-TASSER [26,27,28] server. Input sequences for SULT4A1 (uniprot-id: Q9BR01) and SULT6B1 (uniprot-id: Q6IMI4) originated from uniprot [29]. Only the best model according to the C-score was further considered for both SULT enzymes (SULT4A1: 0.97, SULT6B1: 0.59). A high C-score assumes high model quality confidence and ranges from –5 to 2. The COFACTOR [30,31]/COACH [32] functionality of I-TASSER predicted the coordinates of adenosine-3′,5′-diphosphate (PAP), the depleted form of the cofactor commonly used for co-crystallization with SULTs, for the homology models of the two SULTs. For SULT4A1, COFACTOR/COACH suggests the PAP coordinates in SULT1A1 (PDB-id: 1LS6 [33]) and for SULT6B1 those in mouse SULT1D1 (PDB-id: 2ZPT [34]). For SULT4A1, side chain Tyr91 was rotated outwards of the catalytic pocket to allow for substrate positioning using the Rotamer function in MOE (Molecular Operating Environment 2019. 1; Chemical Computing Group ULC, Montreal, QC, Canada). In a similar fashion, Lys65 and Trp70 in the SULT6B1 model were slightly optimized towards cofactor accommodation.

### 2.11. Substrate Docking Experiments

In order to suggest a binding mode hypothesis, molecular docking experiments of UGT-Glo substrate A (GSA) to the active sites of SULT1E1, SULT2A1, SULT4A1, and SULT6B1 were performed using GOLD [35] (v5.7.0; Genetic Optimization for Ligand Docking; CCDC Software, Cambridge, UK). For SULT1E1 and SULT2A1, the X-ray structure 4JVN [36] of SULT1E1 was used due to the high similarity of the co-crystallized substrate (2,6-dibromo-3-(2,4-dibromophenoxy)phenol) to GSA. For SULT4A1 and SULT6B1 the previously built homology models were used. PAPS was built manually in all three SULT structures based on the PAP coordinates and complexes were prepared using the Structure Preparation functionality in MOE. We performed 25 genetic algorithm (GA) runs at 200% search efficiency using the PLP scoring function. The active site was defined by a sphere with the sulfur atom of PAPS at its center and a radius of 18 Å. The algorithm was instructed to search for diverse solutions (substrate poses) with a root mean square difference (RMSD) of at least 1.5 Å. The obtained docking poses were energy minimized using the MMFF94 force field [37] and visually inspected in LigandScout [38,39,40] (v4.4; Inte:ligand, Vienna, Austria). Plausible and catalytically productive binding was assumed when the hydroxy group of GSA, which undergoes sulfation, formed a hydrogen bond with the catalytic histidine (SULT1E1: His107, SULT2A1: His99, SULT4A1: His111, SULT6B1: His118).

## 3. Results

### 3.1. Strain Construction

For each of the human SULT isoenzymes, the most frequently occurring allozyme was used. Sequences were taken from the NCBI database on 19 June 2018. In the case of more than one allozyme, isoform a was employed. Synthetic DNAs coding for each of the human SULTs were cloned into both the integrative vector pCAD1 [21] and the replicating vector pREP1 [22] to yield 28 new expression plasmids. The host strain NCYC2036 was transformed to uracil prototrophy and leucine auxotrophy by homologous integration of the pCAD1-based clones into the *leu1* locus [25]. These 14 strains were subsequently transformed to leucine prototrophy with the corresponding pREP1-based clones (all strains are listed in Table 1). As consequence, each one of the 14 double-expressor strains contains both an integrated expression unit and an autosomal expression plasmid for the same SULT. The suitability of these double-expressor strains was verified by the whole-cell biotransformation assays performed with YN2 and YN4. The former that bears only the integrated expression unit pCAD1 for SULT2A1, showed no activity towards 7-hydroxycoumarin, but the double-expressor strain YN4, yielded sulfated 7-hydroxycoumarin. Additionally, by this cloning strategy, non-auxotrophic strains are obtained, which facilitates their propagation.

### 3.2. Monitoring of SULT Activity Using Standard Test Substrates

**Figure 1:**
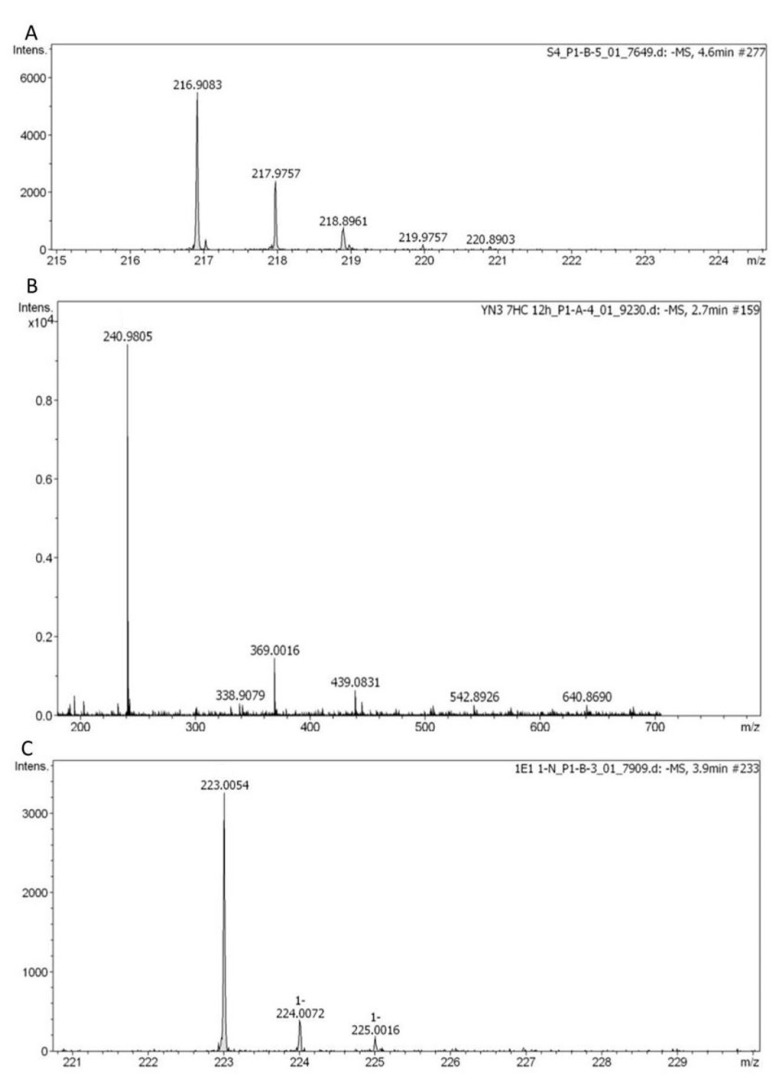
Exemplary results of standard SULT substrates metabolized in whole-cell biotransformations with human SULTs recombinantly expressed in fission yeast. (**A**) 4-nitrophenyl sulfate produced from 4-nitrophenol by SULT1A3 (C_6_H_5_NO_6_S, [M–H]^−^ theor. = 217.9765, [M–H]^–^ exp. = 217.9757, Δ*m*/*z* = 3.67 ppm). (**B**) 7-hydroxycoumarin sulfate produced from 7-hydroxycoumarin by SULT1B1 (C_9_H_6_SO_6_, [M–H]^−^ theor. = 240.9813, [M–H]^–^ exp.= 240.9805, Δ*m*/*z* = 3.32 ppm). (**C**) 1-naphthyl sulfate produced from 1-naphthol by SULT1E1 (C_10_H_8_O_4_S, [M–H]^–^ theor. = 223.0071, [M–H]^–^ exp. = 223.0054, Δ*m*/*z* = 7.62 ppm).

**Figure 2:**
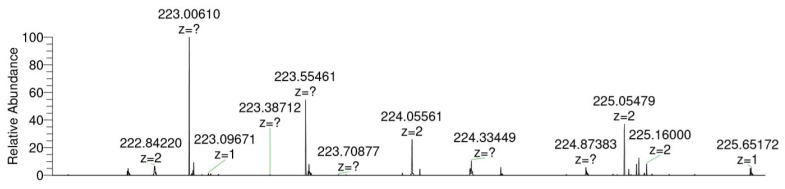
Mass spectrum of 1-naphthyl sulfate produced from 1-naphthol by enzyme bag biotransformation with human SULT4A1 (C_10_H_8_O_4_S, [M–H]^–^ theor. = 223.00705, [M–H]^–^ exp. = 223.00610, Δ*m*/z = 4.26 ppm).

In order to demonstrate the functionality of the human SULTs recombinantly expressed in fission yeast, sulfation activities of the strains expressing one of the twelve human SULT1 or SULT2 family members were tested using known standard substrates. The assay format for these positive control reactions was whole-cell biotransformation. Based on a recent publication on the recombinant expression of several human SULTs in baker’s yeast, which demonstrated that the intracellular level of the cofactor PAPS is sufficiently high for sulfation reactions [6], it was hypothesized that the same might be true for fission yeast. The substrates tested were 4-nitrophenol (for SULT1A2, SULT1A3 SULT1C2, and SULT1C3a), 1-naphthol (for SULT1A1, SULT1C3d, and SULT1E1), 7-hydroxycoumarin (for SULT1B1, SULT1C4, and SULT2A1), and dehydroepiandrosterone (DHEA for SULT2B1a and SULT2B1b), respectively [8,41,42]. Product analysis was done by LC–MS. All SULTs of the families 1 and 2 were found to catalyze the generation of the expected sulfoconjugates. LC–MS chromatograms are shown for SULT1A3, SULT1E1, and SULT1B1, respectively (Figure 1). An additional assay format was developed which employs permeabilized fission yeast cells (enzyme bags) using a protocol similar to those previously reported for CYPs [43] and UGTs [17], but with the addition of the cofactor PAPS instead of NADPH or GSA. In these experiments SULT4A1 catalyzed the sulfation of 1-naphthol (Figure 2), but not of 4-nitrophenol, 7-hydroxycoumarin, or DHEA. To the best of our knowledge, 1-naphthol is therefore the first known substrate for this enzyme. Using SULT6B1, none of these substrates was converted to its sulfate in our assay. Control experiments with the parental strain NCYC2036 were carried out in parallel. In the genome of fission yeast there are no genes with homology to the SULT family and as expected, no formation of any sulfated substrates was seen in control experiments.

### 3.3. Sulfation of a Proluciferin Substrate by the S9 Fraction of Human Liver Cells

**Figure 3:**
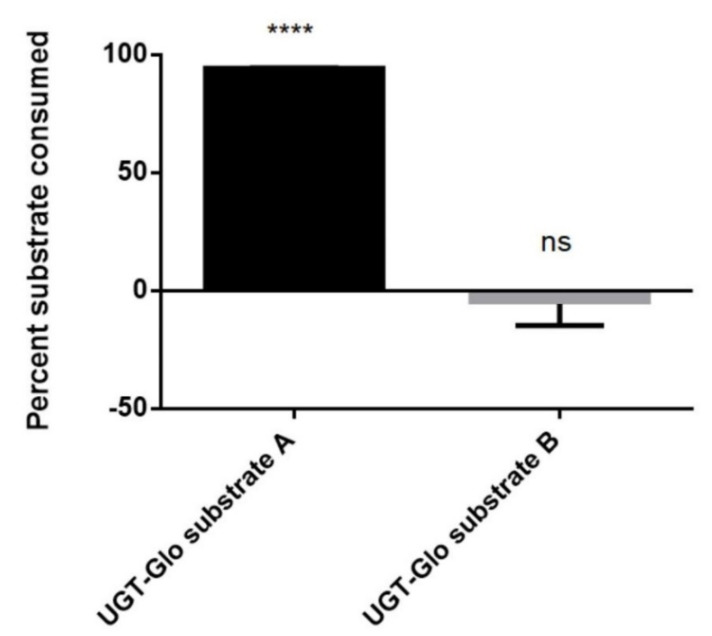
Biotransformation of GSA and GSB by the S9 fraction of human liver cells. Data shown were calculated from two independent experiments. **** *p* < 0.0001 vs. control (i.e., reaction samples without addition of PAPS); n.s.; not significant.

Proluciferin probe substrates for the convenient activity monitoring of CYPs or UGTs have been commercially available for many years. However, a similar test system for the determination of SULT activities was lacking. Since a considerable number of compounds are substrates for both UGTs and SULTs, we speculated that at least one of the two available UGT proluciferin substrates might also be a SULT substrate. In order to test this hypothesis, we performed sulfation reactions using the S9 fraction of human liver cells, the cofactor PAPS, and GSA or GSB. These experiments showed that GSA, but not GSB, is metabolized by the S9 fraction in a PAPS-dependent manner (Figure 3). Formation of sulfated GSA was also confirmed by LC–MS analysis (C_9_H_6_N_2_O_4_S_2_, [M–H]^–^ theor. = 268.96962, [M–H]^–^ exp. = 268.96900, Δ*m*/*z* = 2.31 ppm). The reaction scheme for these experiments is shown in the graphical abstract of this manuscript. In conclusion, these data demonstrate that GSA is a substrate for SULTs contained in the S9 fraction of human liver cells. 

### 3.4. Sulfation of a Proluciferin Substrate by Individual Human SULTs Recombinantly Expressed in Fission Yeast

**Figure 4:**
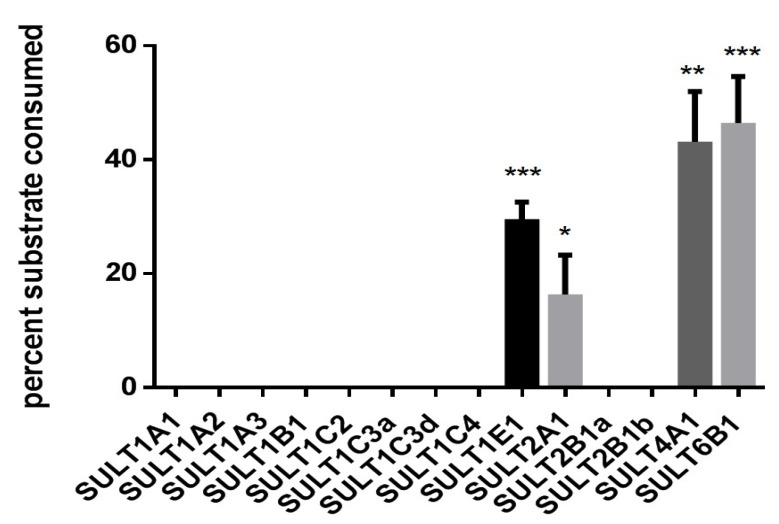
Activity of human SULT enzymes towards GSA. Enzyme bags were prepared from 14 fission yeast strains as indicated and activity was monitored by detecting luminescence. Data shown as percentage of substrate consumed after 3 h of reaction. Data shown were calculated from three independent experiments done in triplicate. * *p* < 0.05; ** *p* < 0.01; *** *p* < 0.001 vs. control (i.e., reaction samples without addition of PAPS).

**Figure 5:**
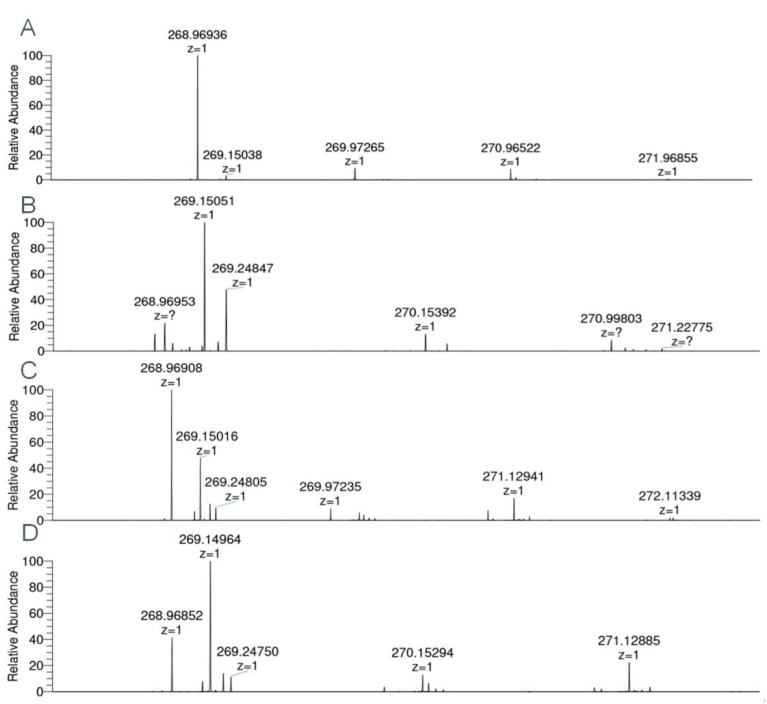
Mass spectra of 6-hydroxy-4-methyl-1,3-benzothiazole-2-carbonitrile sulfate (sulfated GSA) obtained by enzyme bag-catalyzed biotransformations with four human SULTs recombinantly expressed in fission yeast. (**A**) SULT1E1 (C_9_H_6_N_2_O_4_S_2_, [M–H]^−^ theor. = 268.96962, [M–H]^−^ exp. = 268.96936, Δ*m*/*z* = 0.97 ppm). (**B**) SULT2A1 ([M–H]^−^ exp. = 268.96953, Δ*m*/*z* = 0.33 ppm). (**C**) SULT4A1 ([M–H]^−^ exp. = 268.96908, Δ*m*/*z* = 2.01 ppm). (**D**) SULT6B1 ([M–H]^−^ exp. = 268.96852, Δ*m*/*z* = 4.09 ppm).

In order to determine which human SULTs are capable of metabolizing the proluciferin substrate, all 14 fission yeast strains that contain two SULT expression units were tested for metabolization of GSA using the enzyme bag approach. Four of these strains showed a statistically significant substrate sulfation (Figure 4); those were YN25 (expressing SULT1E1), YN4 (SULT2A1), YN32 (SULT4A1), and YN29 (SULT6B1). Formation of sulfated GSA was also confirmed by LC–MS analysis (Figure 5). Together with data from the Genotype-Tissue Expression (GTEx) project, our data suggest that the sulfation of this substrate by S9 fractions is predominantly due to SULT2A1 activity, as expression levels of the other three SULTs in liver are much lower.

### 3.5. Comparative Mechanistic Modeling for SULT1E1, SULT2A1, SULT4A1, and SULT6B1

**Figure 6:**
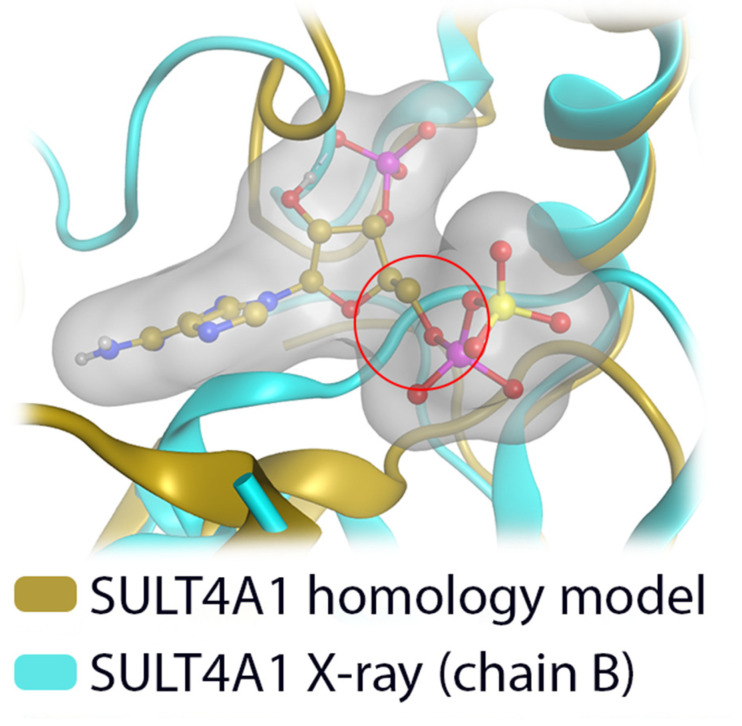
Comparison of our SULT4A1 homology model with the X-ray structure of SULT4A1 (PDB-id: 1ZD1 [8], chain B). The homology model can accommodate the cofactor PAPS, while in the X-ray structure it clashes with the protein backbone (red circle).

**Figure 7:**
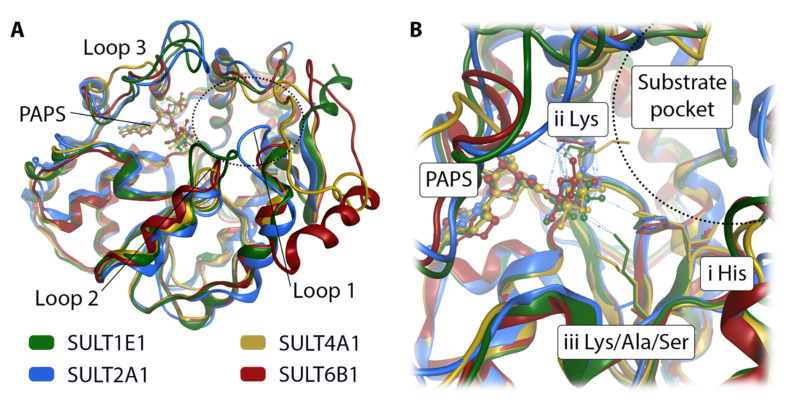
Superposition of four SULT isoforms with cofactor PAPS. SULT1E1 and SULT2A1 are X-ray structures taken from the PDB. SULT4A1 and SULT6B1 are homology models. (**A**) The view of the full structures shows deviations in three loops contributing to the binding site. (**B**) A closer look at the catalytic site shows the position of the conserved catalytically relevant histidine (i His). A lysine (ii Lys) is located in the vicinity of the substrate pocket (indicated by a dotted semicircle), while the second lysine is only present in SULT1E1 and SULT4A1 (iii Lys).

**Figure 8:**
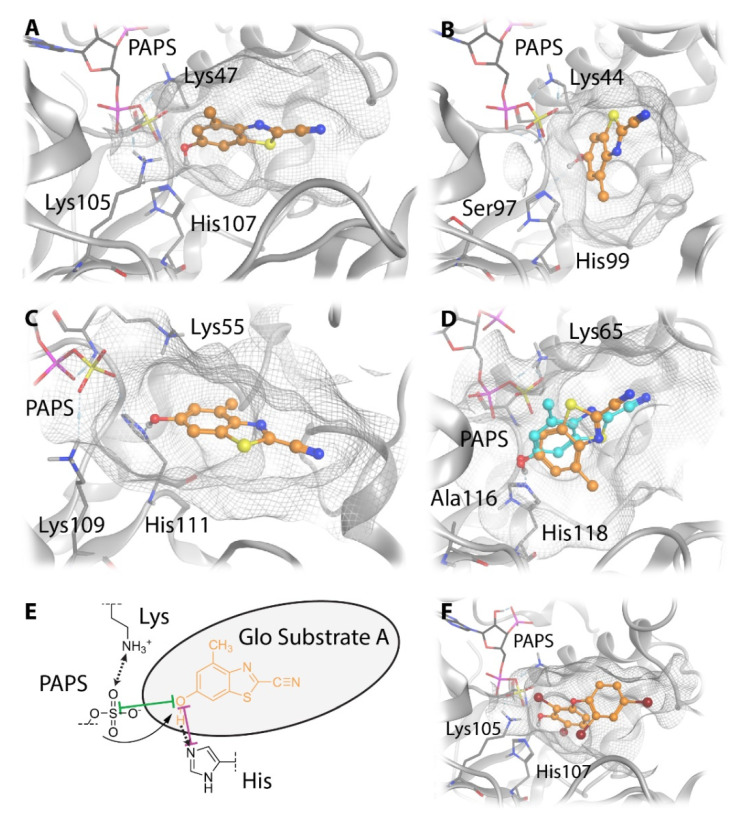
Suggested binding modes of GSA to four different SULT isoforms. (**A**–**D**) The most plausible docking poses of GSA to SULT1E1 (**A**), SULT2A1 (**B**), SULT4A1 (**C**), and SULT6B1 (**D**). (**E**) Distances of the hydroxy group of GSA to the catalytic histidine and the cofactor PAPS are displayed in a 2D substrate pocket depiction. (**F**) The X-ray binding mode of the co-crystallized ligand 2,6-dibromo-3-(2,4-dibromophenoxy) phenol to SULT1E1 is shown for comparison (PDB-id: 4JVN).

In order to rationalize the reported activity of GSA in SULT1E1, SULT2A1, SULT4A1, and SULT6B1, respectively, we performed mechanistic molecular modeling of the four respective enzyme substrate complexes. For SULT1E1 and SULT2A1 the available X-ray structures were used. The only available X-ray structure of SULT4A1 (PDB-id: 4JVN [36]) cannot accommodate the cofactor PAPS (Figure 6), and SULT6B1 has no solved X-ray structure. Hence, we designed homology models of SULT4A1 and SULT6B1 using the I-TASSER [26,27,28] server. A structural comparison of the four isoforms reveals that the backbone position is highly similar except for three loop regions that surround the substrate pocket (Figure 7A). High structural flexibility has been reported for loops 2 and 3 in SULT1E1 [44]. There are three prominent residue positions in the catalytic pocket (Figure 7B): Firstly, the conserved histidine crucial for the deprotonation of the substrate’s hydroxy moiety (Figure 7B and Figure 8E). Secondly, a lysine facilitating the sulfation of the substrate by holding the cofactor PAPS in place (Figure 7B and Figure 8E), explaining its conservation. The residue in the third position is not conserved. In SULT1E1 (Figure 8A) and SULT4A1 (Figure 8C) the lysine at the third position likely interacts with PAPS and the substrate, thereby assisting the reaction. Ser97 in SULT2A1 (Figure 8B) and Ala116 in SULT6B1 (Figure 8D) in the same position cannot adopt this functionality. This does not seem to decrease the sulfation rate of GSA in these two SULT isoforms (Figure 4). According to our homology model of SULT4A1, the rest of the active site comprises residues Glu14, Leu27, Pro28, Pro29, Phe30, Cys31, Pro54, Lys55, Val88, Glu90, Tyr91, Pro92, Lys109, His111, Tyr142, Phe145, Thr151, Met152, Gly171, and Tyr172. Surprisingly, the model of catalytically competent SULT4A1 resembled SULT1B1 (PDB-id: 3CKL) more than the X-ray structure of SULT4A1 (PDB-id: 1ZD1 [8]) according to TM-align [45], a program implemented in I-TASSER. The substrate pocket of SULT6B1 consists of the following further residues: Met39, Ser68, Tyr89, Phe92, Val94, Glu96, Cys97, Gly98, His118, Phe152, Pro157, Asp158, Trp179, His250, Val253, and Leu257, as suggested by the homology model.

However, the catalytic histidine and cofactor-binding lysine are highly conserved in all four SULT isoforms, and differences in the third key residue position do not rationalize the observed activity trend of UGT-Glo substrate A. Hence, we docked this substrate to the active sites of the four SULT isoforms to suggest differences in the binding modes. The most plausible orientations of the substrate from docking (Figure 8A–D) resemble the reference binding mode of the co-crystallized ligand in the SULT1E1 X-ray structure (Figure 8F, PDB-id: 4JVN). The slot-like substrate pockets orient the substrate in a horizontal position in SULT1E1 (Figure 8A) and more vertically in SULT2A1 (Figure 8B). The substrate pockets of SULT4A1 (Figure 8C) and SULT6B1 (Figure 8D) are more voluminous and open. In SULT6B1, GSA can even adapt two distinct catalytically competent orientations.

In all SULTs a hydrogen bond is formed between the substrate hydroxy group and the catalytic histidine that is crucial for the reaction (Figure 8E). The docking poses suggest a difference in the distance and therefore in the quality of the hydrogen bond (Table 2). We suggest that the apparent different orientations of GSA in the SULT isoforms are caused by the different substrate pocket shapes. The less restrictive pockets in SULT4A1 and SULT6B1 allow for stronger hydrogen bonding, as suggested by a lower O_GSA_-N_His_ atom distance. The increased O_GSA_–S_PAPS_ atom distance can likely be reduced as the cofactor adopts a more favorable orientation towards the substrate.

## 4. Discussion

A set of fission yeast strains was created that contain expression constructs for each of the human SULT genes (Table 1). Sulfation activities of the twelve strains expressing human SULT1 or SULT2 enzymes were confirmed by whole-cell biotransformations using the known standard substrates 4-nitrophenol (for SULT1A2, SULT1A3 SULT1C2, and SULT1C3a), 1-naphthol (for SULT1A1, SULT1C3d, and SULT1E1), 7-hydroxycoumarin (for SULT1B1, SULT1C4, and SULT2A1), and DHEA (for SULT2B1a and SULT2B1b), respectively (exemplary results in Figure 1). These results confirm that the intracellular level of the cofactor PAPS is sufficiently high for sulfation reactions in fission yeast, as was previously demonstrated in similar experiments in baker’s yeast [6].

Recently, we reported the successful use of permeabilized fission yeast cells (enzyme bags) for biotransformations catalyzed by human CYPs [43] or UGTs [17]. Such an assay design has the advantages of higher sensitivity and shorter reaction times, as substrates, cofactors, and products are not hampered by various biological membranes but can freely move between the assay medium and the inside of the cells. For comparison, the activity of SULT1C3d towards the standard substrate 1-naphthol was monitored with both enzyme bags and whole-cell biotransformation assays. Whole-cell assay resulted in no detection of the sulfated substrate, while in enzyme bags the sulfation of 1-naphthol was confirmed, which underlined the efficiency of enzyme bags assay. On the downside, the (sometimes expensive) cofactors need to be supplied to the reaction mixture. Thus, enzyme bag assays are well suited for enzymatic studies, whereas whole-cell biotransformations are to be preferred for (large scale) metabolite productions as they are cheaper and can be upscaled much easier. However, in contrast to membrane-bound human CYPs and UGTs, human SULTs are soluble proteins and might therefore be washed out of enzyme bags more easily. In our original study on the generation of enzyme bags from recombinant fission yeast cells, we demonstrated that upon permeabilization with 0.3% (*v*/*v*) Triton X-100, endogenous glucose 6-phosphate dehydrogenase (G6PDH) activity levels remain unaffected while small molecules can easily pass into and out of the cells [46]. There are three putative enzymes with G6PDH activity in fission yeast (gcd1, zwf1, and SPAC3C7.13c), which have sizes between 54 and 57 kDa [47]; thus, soluble proteins of this size (and possibly also slightly smaller ones) are expected to remain inside the enzyme bags. With the possible exception of SULT6B1, human SULTs exist as dimers of 61 to 82 kDa [2], so their size is expected to be compatible with the enzyme bag approach. We therefore tested this assay format using SULT-expressing fission yeast strains and the cofactor PAPS. We could not only show that this method is applicable for human SULTs, but we even observed activity of SULT4A1 towards 1-naphthol (Figure 2), thereby demonstrating the first enzymatic activity for this enzyme. Additionally, GSA was found to be successfully converted to its sulfate utilizing SULT4A1 and SULT6B1.

In general, probe substrates for drug metabolizing enzymes are compounds that are efficiently converted into readily detectable products. Detection methods that are used to monitor such conversions include light or UV absorbance, fluorescence, mass spectrometry, radiometry, and bioluminescence [48]. With respect to the latter, many proluciferin probe substrates are available which are (more or less selectively) metabolized by certain CYPs or UGTs to reaction products that emit photons upon a second reaction step catalyzed by luciferase. However, a similar test system for the determination of SULT activities was not yet described. Since there is a significant overlap in the substrate selectivities of the human UGT and SULT families, it was reasonable to assume that at least one of the two available UGT proluciferin substrates may also be metabolized by SULTs. Sulfation reactions using these probe substrates together with the S9 fraction of human liver cells indeed demonstrated that GSA, but not GSB, is converted in a PAPS-dependent manner (Figure 3). Individual testing of the recombinant fission yeast strains showed that this reaction is catalyzed by SULT1E1, SULT2A1, SULT4A1, and SULT6B1 (Figure 4 and Figure 5). Thus, a new probe reaction for these four enzymes was identified. This is especially noteworthy for SULT4A1 and SULT6B1, as its availability is expected to aid in the search for physiological substrates of these two enzymes. In addition, our data suggest that GSA sulfation by S9 fractions of human liver cells is mainly due to SULT2A1 activity.

In order to investigate the GSA activity trends, we constructed catalytically competent homology models for SULT4A1 and SULT6B1 with the novelty of PAPS cofactor accommodation (Figure 6 and Figure 7). Structural comparison of the four GSA-metabolizing SULT isoforms identified different active site shapes. In SULT1E1 and SULT2A1, the substrate pocket is more slot-like, whereas it is more voluminous in SULT4A1 and SULT6B1. Molecular docking of GSA suggests that the less restrictive active sites in SULT4A1 and SULT6B1 enhance substrate binding and thereby increase its sulfation rate (Figure 8). The docking poses suggest varying lengths of the catalytically important hydrogen bond formed between the substrate hydroxy group and the catalytic histidine (Table 2), which are likely due to the different substrate pocket shapes of these four enzymes.

Taken together, in this study we show that all 14 human SULTs may be functionally expressed in fission yeast *S. pombe*. Moreover, we demonstrate for the first time that SULT4A1 and SULT6B1 are indeed catalytically active enzymes and we present test substrates for each of them. It is expected that this knowledge will contribute to the identification of physiologically important substrates of these enzymes and will thus contribute to elucidating their functions.

## Figures and Tables

**Table 1:** List of fission yeast strains used in this study.

Strain	Parental Strain	Expressed Proteins	Genotype	Reference
NCYC2036	None	None	h- ura4-D.18	[25]
YN5	NCYC2036	SULT1A1	h- ura4-D.18 leu1::pCAD1- SULT1A1	This study
YN6	NCYC2036	SULT1A2	h- ura4-D.18 leu1::pCAD1- SULT1A2	This study
YN7	NCYC2036	SULT1A3	h- ura4-D.18 leu1::pCAD1- SULT1A3	This study
YN1	NCYC2036	SULT1B1	h- ura4-D.18 leu1::pCAD1- SULT1B1	This study
YN8	NCYC2036	SULT1C2	h- ura4-D.18 leu1::pCAD1- SULT1C2	This study
YN9	NCYC2036	SULT1C3a	h- ura4-D.18 leu1::pCAD1- SULT1C3a	This study
YN10	NCYC2036	SULT1C3d	h- ura4-D.18 leu1::pCAD1- SULT1C3d	This study
YN11	NCYC2036	SULT1C4	h- ura4-D.18 leu1::pCAD1- SULT1C4	This study
YN12	NCYC2036	SULT1E1	h- ura4-D.18 leu1::pCAD1- SULT1E1	This study
YN2	NCYC2036	SULT2A1	h- ura4-D.18 leu1::pCAD1- SULT2A1	This study
YN13	NCYC2036	SULT2B1a	h- ura4-D.18 leu1::pCAD1- SULT2B1a	This study
YN14	NCYC2036	SULT2B1b	h- ura4-D.18 leu1::pCAD1- SULT2B1b	This study
YN17	NCYC2036	SULT4A1	h- ura4-D.18 leu1::pCAD1- SULT4A1	This study
YN15	NCYC2036	SULT6B1	h- ura4-D.18 leu1::pCAD1- SULT6B1	This study
YN18	YN5	SULT1A1 (twice)	h- ura4-D.18 leu1::pCAD1-SULT1A1/pREP1-SULT1A1	This study
YN19	YN6	SULT1A2 (twice)	h- ura4-D.18 leu1::pCAD1- SULT1A2/pREP1-SULT1A2	This study
YN20	YN7	SULT1A3 (twice)	h- ura4-D.18 leu1::pCAD1- SULT1A3/pREP1-SULT1A3	This study
YN3	YN1	SULT1B1 (twice)	h- ura4-D.18 leu1::pCAD1- SULT1B1/ pREP1-SULT1B1	This study
YN21	YN8	SULT1C2 (twice)	h- ura4-D.18 leu1::pCAD1- SULT1C2/pREP1-SULT1C2	This study
YN22	YN9	SULT1C3a (twice)	h- ura4-D.18 leu1::pCAD1- SULT1C3a/pREP1-SULT1C3a	This study
YN23	YN10	SULT1C3d (twice)	h- ura4-D.18 leu1::pCAD1- SULT1C3d/pREP1-SULT1C3b	This study
YN24	YN11	SULT1C4 (twice)	h- ura4-D.18 leu1::pCAD1- SULT1C4/pREP1-SULT1C4	This study
YN25	YN12	SULT1E1 (twice)	h- ura4-D.18 leu1::pCAD1- SULT1E1/pREP1-SULT1E1	This study
YN4	YN2	SULT2A1 (twice)	h- ura4-D.18 leu1::pCAD1- SULT2A1/pREP1-SULT2A1	This study
YN31	YN13	SULT2B1a (twice)	h- ura4-D.18 leu1::pCAD1- SULT2B1a/pREP1-SULT2B1a	This study
YN27	YN14	SULT2B1b (twice)	h- ura4-D.18 leu1::pCAD1- SULT2B1b/pREP1-SULT2B1b	This study
YN32	YN17	SULT4A1(twice)	h- ura4-D.18 leu1::pCAD1- SULT4A1/pREP1-SULT4A1	This study
YN29	YN15	SULT6B1 (twice)	h- ura4-D.18 leu1::pCAD1- SULT6B1/pREP1-SULT6B1	This study

**Table 2:** Distances of the hydroxy moiety of GSA in the binding modes suggested by docking.

SULT Isoform	Substrate Pose	d(O_GSA_-N_His_) ^b^[Å]	d(O_GSA_-S_PAPS_) ^b^[Å]
SULT1E1	X-ray ^a^	3.0	3.3
SULT1E1	1	3.4	3.6
SULT2A1	1	3.4	3.8
SULT4A1	1	2.8	4.2
SULT6B1	1	2.5	4.7
SULT6B1	2	2.6	5.0

^a^ Reference; ^b^ distance between indicated atoms.
